# Validation of the RunScribe inertial measurement unit for walking gait measurement

**DOI:** 10.1371/journal.pone.0273308

**Published:** 2022-08-22

**Authors:** Max Lewin, Carina Price, Christopher Nester

**Affiliations:** School of Health and Society, University of Salford, Manchester, United Kingdom; Ningbo University, CHINA

## Abstract

**Introduction:**

The use of portable gait measurement systems in research is appealing to collect real-world data at low-cost, low participant burden, and without requirement for dedicated lab space. Most commercially available inertial measurement units (IMU’s) designed for running only capture temporospatial data, the ability to capture biomechanics data such as shock and motion metrics with the RunScribe IMU makes it the closest to a lab alternative. The RunScribe system has been validated in running, however, is yet to be validated for walking.

**Method:**

Qualisys motion capture, AMTI force plates, and Delsys Trigno accelerometers were used as gold standard lab measures for comparison against the RunScribe IMU. Twenty participants completed 10 footsteps per foot (20 total) measured by both systems simultaneously. Variables for validation included: Vertical Ground reaction force (GRF), instantaneous GRF rate, pronation excursion, pronation velocity, total shock, impact force, braking force. Interclass correlation (ICC) was used to determine agreement between the measurement systems, mean differences were used to evaluate group level accuracy.

**Results:**

ICC results showed moderate agreement between measurement systems when both limbs were averaged. The greatest agreement was seen for GRF rate, pronation excursion, and pronation velocity (ICC = 0.627, 0.616, 0.539), low agreement was seen for GRF, total shock, impact shock, braking shock (ICC = 0.269, 0.351, 0.244, 0.180). However mean differences show the greatest level of accuracy for GRF, GRF rate, and impact shock.

**Discussion:**

Results show mixed agreement between the RunScribe and gold standard lab measures, and varied agreement across left and right limbs. Kinematic variables showed the greatest agreement, however GRF had the lowest relative mean difference for group results. The results show acceptable levels of agreement for most variables, however further work must be done to assess the repeatability and sensitivity of the RunScribe to be applied within areas such as footwear testing and gait retraining protocols.

## Introduction

Gait studies were traditionally undertaken in gait laboratories that require a dedicated space and expensive and technically complex measurement devices including motion capture, force platforms, accelerometers, and electromyography (EMG) as key examples. The possibility to collect data in real-world contexts is attractive because it avoids some of these challenges, especially given the impact of the covid-19 pandemic on research facilities. It also avoids other important pitfalls of laboratory-based gait studies. Laboratory studies can be a burden to participants who need to be in a set location at a specific date/time which can be a disincentive to participation. It also avoids the influence that researcher presence is known to have on the validity of natural gait during lab-based data collection [1]. Finally, laboratory data requires post-processing which can be time consuming and delay access to results and slow subsequent decision making.

Gait data collected outside of a laboratory setting has arguably far greater external validity as it allows for a more natural gait to be captured and continuous data collection over a longer period of time than laboratory studies allow [2]. The opportunity therefore arises for studies that once took place in laboratory settings to now be undertaken with greater external validity through use of portable measurement systems, as long as such systems accurately measure the pertinent variables. Testing of footwear and foot orthoses in terms of their impact on ground reaction forces, foot motion, especially pronation, and “shock”, have occupied many researchers where a holistic approach of gait characterisation commonly requires laboratory-based study [3, 4]. The ability to transfer objective orthotic testing from a lab into the real world is therefore attractive, with many factors working in the favour of portability, including cost, space, availability, and application within participants.

This notion has led to development of hardware enabling out of lab data collection, with development of portable systems for gait measurement [5] and measurement of temporospatial gait parameters on different outdoor surfaces [6]. This has facilitated data collections such as the comparison of gait in healthy individuals and Parkinson’s patients whereby participants used measurement systems in their daily life for 7 days [7]. Many wearable sensor systems focus on measurement of temporospatial gait parameters, targeted towards recreational runners and their running performances and habits (e.g. ARION, NURVV, Stryd, GWalk, RunScribe). Common gait parameters include step length, stride length, contact time, speed, pace, distance, and duration. Previous study has shown good levels of agreement for the Stryd (ICC > 0.81) when compared to high-speed video for contact time, flight time, step frequency, and step length during running [8]. Comparison against an OptoGait gait measurement system at different running speeds showed high ICC values for step length (>0.934) and step frequency (>0.956), however agreement was lower for contact time (<0.463) and flight time (0.555–0.806) [9]. When compared to a motion capture system, measures of ground contact time and leg spring stiffness measured by the Stryd were deemed acceptable [10]. The GWalk has shown good reliability for speed, cadence, stride duration and stride length (rho > 0.75) in comparisons against an instrumented carpet [11], and high ICC values (> 0.728) have also been seen in test-retest reliability analyses of a range of gait parameters measured by the GWalk [12]. The RunScribe IMU perhaps provides the most in-depth analysis of all these units, offering measurement including pronation excursion, pronation velocity, impact shock, braking shock, total shock ground reaction force (GRF), and GRF rate. Validation studies have only focused on pronation variables at running speeds where agreement with a 3D motion system was very mixed (Pronation Excursion (ICC = 0.4–0.57), Pronation Velocity (ICC = 0.74–0.87)) [13], and running shock in different footwear conditions with low correlation between the RunScribe and tibial accelerometer (r = 0.42) and between the RunScribe and a shoe mounted accelerometer (r = 0.57) [14]. A more inferential study demonstrated that the RunScribe shock variables were significantly different between surfaces (P = 0.001) and speeds (P<0.001) indicating the ability for the RunScribe to detect change between conditions [15].

The RunScribe IMU is the most rounded wearable gait measurement device with a range of biomechanical variables relevant for human gait measurement including pronation excursion, pronation velocity, ground reaction force (GRF), GRF rate, impact shock, braking shock, and total shock in addition to temporospatial gait parameters. Pronation and shock variables are measured directly by the RunScribe unit and system, however GRF parameters are estimated using equations utilising contact time and flight time measured by the RunScribe, these equations were defined in previous research on middle distance runners [16]. In comparison to other units, the RunScribe provides measurement of variables pertinent to footwear and orthotic testing, and the design of orthoses. Therefore validating the RunScribe IMU to ensure the unit provides accurate and reliable measures of these variables during walking would enable the advancement of externally valid testing of footwear and orthotic products. Footwear testing that once took place in laboratory settings could be undertaken in real-world settings with greater external validity.

### Aims & hypothesis

The aims of the current study were to assess the accuracy of the RunScribe IMU against Gold standard biomechanics laboratory-based measurements of the data it provides on ground reaction forces, foot kinematics, and shock. It is hypothesised that agreement will be mixed across all variables and that pronation and shock variables will show the greatest level of agreement to the gold standard lab measures due to the direct measurement of these variables, agreement will be lower for force variables due to inference of these variables. However, methodological constraints may reduce accuracy of the shock variables as measured by the RunScribe system compared to the laboratory-based measurement of the shock variables due to the placement of the RunScribe on the foot, and the laboratory sensor on the shank.

## Methods

Twenty Participants (Male n = 8, Female n = 12) took part in the testing: Age (33.6 ± 10.6 Years), Height (170.9 ± 7.8 cm), Body Mass (73.2 ± 11.9 Kg). Ethical clearance for the protocol was granted by The University of Salford ethics committee, application number 1391. Written consent was obtained from all participants prior to commencement of the research protocol.

### Gold standard lab measurements

**Fig 1:**
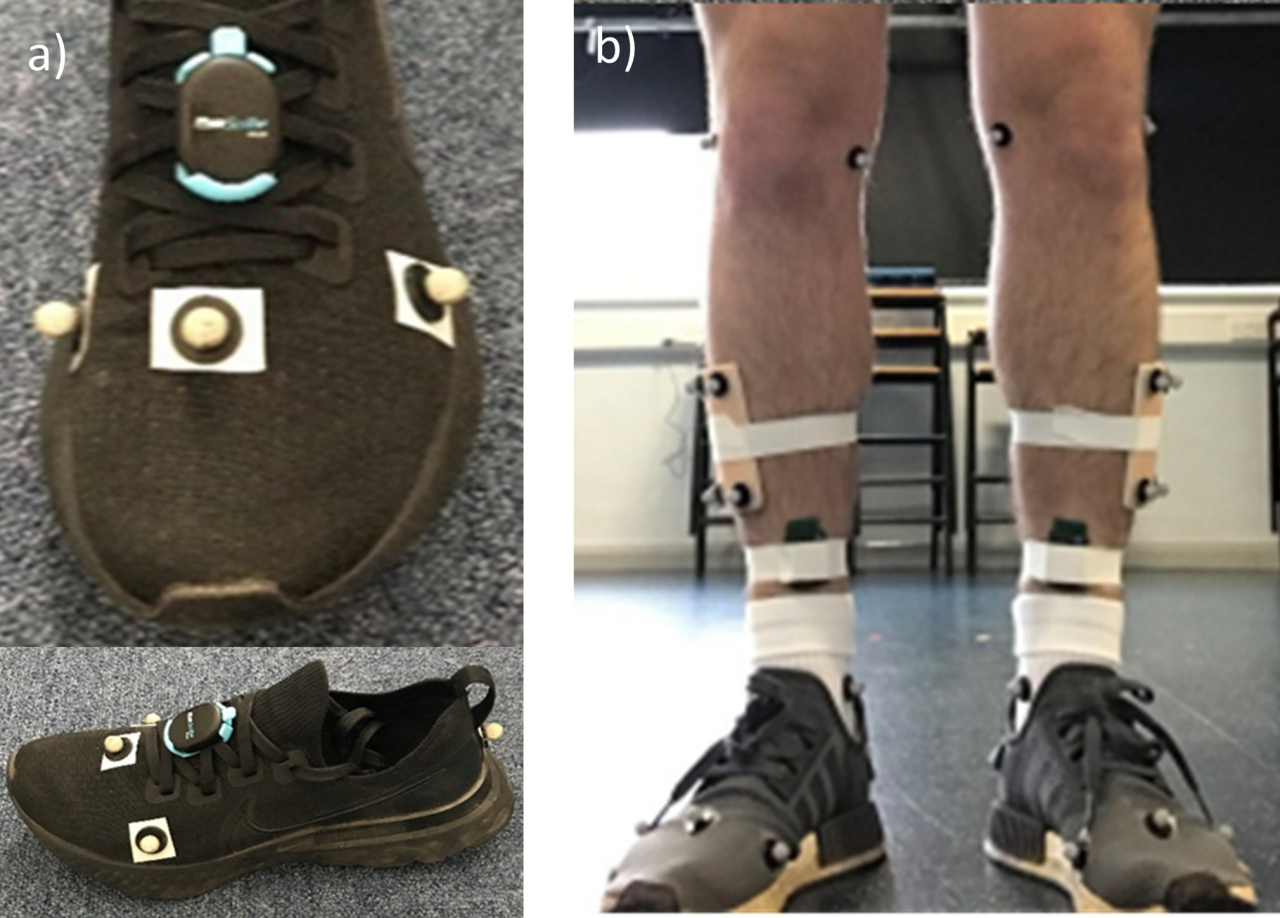
Marker and equipment placement on a) Participant footwear and b) lower limbs.

Participants walked at self-selected speed across a laboratory walkway whilst foot kinematics, GRF and accelerometer data were collected simultaneously. Two instrumented force plates (AMTI, Massachusetts, USA) operating at 1000 Hz were spaced to allow both plates to be contacted with the same foot. Thirteen Qualisys Oqus cameras (Qualisys, Gothenburg, Sweden) operating at 100 Hz were used for 3D motion capture. Marker setup was completed bilaterally as follows: Medial knee, Lateral Knee, Medial Malleolus, Lateral Malleolus, Heel, MH1, MH2, MH5, with the addition of a 4 marker cluster on the outer shank (Fig 1). A Delsys Trigno Avanti (Delsys, Natick, Massachusetts, USA) unit sampling at 135Hz was affixed bilaterally to the shank of participants and subsequently wrapped using medical tape to fix the accelerometer in place and prevent movement during the protocol. Participants completed 5 walks targeting the force plate with the left foot, and 5 targeting with the right foot, resulting in a total of 10 steps per foot for comparison. Participants completed this whilst wearing their own footwear.

Data from force plate contact only was processed using Visual 3D (C-Motion, Maryland, USA) to extract GRF (xBW) and peak GRF rate (N/s) from force plate data, and Pronation Excursion (°), Maximum pronation velocity (°/sec) from marker-based motion data. Pronation Excursion was defined as the amount of pronation from initial contact to maximum pronation, maximum pronation velocity was the maximum instantaneous velocity of joint rotation during this period. A Butterworth low pass filter was used on marker trajectories (10Hz) and force data (20Hz). The shank was modelled using medial and lateral knee markers, shank cluster and medial and lateral malleolus markers. The foot was modelled as a whole using medial and lateral malleolus, heel, and metatarsal head markers. Pronation was defined as the rotation of the foot with respect to the shank in the frontal plane. Peak positive vertical acceleration and peak negative horizontal acceleration was taken from accelerometer data as the impact shock (g) and braking shock (g) respectively and combined in the same manner as the RunScribe to give total shock (g).

### RunScribe IMU

The RunScribe Plus IMU device (500 Hz) (Scribe labs, Moss Beach, California, USA) was fitted to the laces of both left and right shoes as per the manufacturer instructions (Fig 1). The RunScribe is a commercially available device with a pre-programme algorithm that delivers maximum values of the variables in question per step, rather than delivering the data across the whole time of the gait cycle. The RunScribe system was started manually before each walk across the force plate, and stopped when the individual had passed the force plates. This enabled the steps before and after the steps with force plate contact to be captured by the RunScribe. Individuals approached the force plate in a self-selected manner, the number of steps before the force plate was identified and these steps were used to identify the next two steps as the steps that contacted the force plate. The data could then be extracted from the RunScribe for the relevant steps that contacted the force plates, and then matched to the data for the same footsteps from the gold standard laboratory system post processing. Data was downloaded and the steps matching those used for GRF, kinematic, and acceleration data collection extracted. Variables taken from the RunScribe IMU are detailed in Table 1.

**Table 1:** Definition of variables measured by the RunScribe Plus IMU.

Variable	RunScribe definition
Pronation Excursion (°)	Amount of rotation from initial foot contact to maximum pronation
Pronation Velocity (°/sec)	Maximum velocity of pronation between initial foot contact and maximum pronation
GRF (xBW)	Peak vertical GRF
GRF Rate (N/Kg/s)	Mean vertical force during stance
Total Shock (g)	Vector combination of impact and braking shock
Impact Shock (g)	Peak positive vertical acceleration
Braking Shock (g)	Peak negative horizontal acceleration

Data from all systems was collected on the left and right limbs, and a single measure per participant was also created through averaging these scores. All data was included for further analysis to provide insight into repeatability through the differences between the systems in both limbs. The average measure was created to provide a single reference measure for comparison of the two measurement systems.

### Statistics

SPSS statistics 26 (IBM, New York, USA) was used to conduct a two-way mixed effects ICC with average measures and absolute agreement (ICC 3,1) to determine the level of agreement between the RunScribe IMU (RS) and the gold standard lab measures (LAB), the test was completed on left and right data individually, and the averaged data. ICC values are classed as: <0.5 (low), 0.5–0.75 (moderate), 0.75–0.9 (good), 0.9–1.0 (excellent) [17]. Mean differences were calculated as LAB minus RS, percentage mean difference was calculated using these mean differences as a percentage of the lab value. Outlying data was consistently present for 2 participants for the GRF rate, and all 3 shock variables. These outliers caused the data to be non-normally distributed, the outlying data was subsequently explored however there was no evident reasoning for the outlying data, therefore these outliers were removed from analysis leaving data normally distributed.

## Results

Data comparing the outcome variables averaged for both limbs is present in Table 2. Moderate levels of agreement between RS and LAB are present for pronation variables. Agreement was low for GRF (0.269), but moderate for GRF rate (0.627). There was also low agreement for all shock variables. Mean differences show GRF, impact shock, and GRF rate to be comparable across systems with differences of less than ±4%. Greater mean differences are present for braking shock, total shock, pronation excursion, and pronation velocity with differences from ± 20.06–88.90%

**Table 2:** Comparison of all variables with data from both limbs combined (Mean ± SD), with ICC and mean differences (LAB–RS).

Variable	RS	LAB	ICC	Mean Difference	% Mean Difference
Pronation Excursion (°)	8.05 ± 3.78	10.07 ± 1.90	0.616	2.02 ± 2.88	20.06
Pronation velocity (°/sec)	229.82 ± 70.16	182.15 ± 41.04	0.539	-47.67 ± 58.22	-26.17
GRF (xBW)	1.16 ± 0.05	1.13 ± 0.04	0.269	-0.03 ± 0.06	-2.62
GRF rate (N/s)	14.12 ± 0.84	14.66 ± 2.40	0.627	0.54 ± 1.87	3.65
Total shock (g)	2.54 ± 0.41	1.79 ± 0.47	0.351	-0.76 ± 0.43	-42.30
Impact shock (g)	1.47 ± 0.37	1.42 ± 0.42	0.244	-0.06 ± 0.52	-3.92
Braking shock (g)	1.94 ± 0.39	1.03 ± 0.37	0.180	-0.92 ± 0.42	-88.90

Data the outcome variables for the left foot is present in Table 3. Moderate levels of agreement were again seen between the two measurement systems for pronation variables. Agreement was low for GRF (0.383) and moderate for GRF rate (0.594). Agreement was again low but close to moderate for total shock (0.434) and impact shock (0.474), however was low for braking shock (0.269). Data was comparable across systems for GRF rate, GRF, and impact shock with mean differences less than ± 3%. Mean differences from ± 15.76 to 78.10% were present for braking shock, pronation excursion, pronation velocity, and total shock.

**Table 3:** Comparison of left foot data from all variables (Mean ± SD), with ICC and mean differences (LAB—RS).

Variable	RS	LAB	ICC	Mean Difference	% Mean Difference
Pronation Excursion (°)	8.29 ± 4.53	9.84 ± 2.07	0.606	1.55 ± 3.64	15.76
Pronation velocity (°/sec)	221.26 ± 74.76	177.97 ± 40.62	0.510	-43.29 ± 64.72	-24.33
GRF (xBW)	1.16 ± 0.07	1.13 ± 0.04	0.383	-0.03 ± 0.06	-2.74
GRF rate (N/s)	14.13 ± 0.91	14.44 ± 2.66	0.594	0.31 ± 2.15	2.14
Total shock (g)	2.53 ± 0.54	1.86 ± 0.55	0.434	-0.68 ± 0.55	-36.40
Impact shock (g)	1.53 ± 0.40	1.49 ± 0.46	0.474	-0.04 ± 0.51	-2.69
Braking shock (g)	1.88 ± 0.55	1.06 ± 0.43	0.269	-0.83 ± 0.56	78.10

Data comparing systems for the right foot is present in Table 4. Agreement was again moderate in the right limb for pronation variables. Very low agreement was seen for GRF (0.092), and moderate agreement for GRF rate (0.579). Low agreement was again seen for all shock variables (ICC < 0.260). Mean differences show GRF, impact shock, and GRF rate to be comparable across systems with differences less than ±6%. Mean differences show less comparable results between systems for pronation excursion, pronation velocity, total shock, and braking shock with differences from ± 24.16 to 100.32%.

**Table 4:** Comparison of right foot data from all variables (Mean ± SD), with ICC and mean differences (LAB—RS).

Variable	RS	LAB	ICC	Mean Difference	% Mean Difference
Pronation Excursion (°)	7.82 ± 3.65	10.31 ± 2.48	0.604	2.49 ± 2.92	24.16
Pronation velocity (°/sec)	238.37 ± 76.66	186.32 ± 52.34	0.600	-52.05 ± 62.00	-27.94
GRF (xBW)	1.15 ± 0.05	1.12 ± 0.04	0.092	-0.03 ± 0.06	-2.49
GRF rate (N/s)	14.10 ± 0.81	14.87 ± 2.42	0.579	0.76 ± 1.92	5.12
Total shock (g)	2.55 ± 0.56	1.71 ± 0.46	0.260	-0.84 ± 0.58	-48.70
Impact shock (g)	1.42 ± 0.47	1.35 ± 0.41	0.189	-0.07 ± 0.59	-5.28
Braking shock (g)	2.00 ± 0.51	1.00 ± 0.39	0.115	-1.00 ± 0.57	-100.32

## Discussion

The current study is the first to attempt to validate the RunScribe IMU for use in walking. The comparison to gold standard lab shows mixed levels of agreement. The RunScribe both under-estimated and over-estimated variables, however differences apparent within the left and right limbs were always unidirectional. Overall agreement was moderate with some variables with low agreement, kinematic variables showed greater levels of agreement than all other variables. The mean differences also show mixed results, with large relative mean differences for braking shock and low mean differences in GRF. Mean differences also displayed inconsistency within some variables with large differences between left and right limbs.

GRF data consistently shows the lowest mean difference (-2.49 –-2.74%) of all variables, although agreement was low (ICC = 0.092–0.383). Low relative mean differences were also present for the GRF rate data (2.14%– 5.12%), however with greater, moderate levels of agreement (ICC = 0.579–0.627). There were no previous studies attempting to validate these variables as measured by the RunScribe in either walking or running. The GRF data is the only data measured by the RunScribe within the study that is inferred as opposed to directly measured. This may provide evidence for the low levels of agreement seen within these variables. However, the Mean differences were the lowest of all variables within the study. Within lab studies GRF is measured using instrumented force plates, IMU based systems with similar portability of the RunScribe are available for estimates of GRF but do not provide a direct measurement of GRF. Systems such as the TekScan F-Scan are available for portable measures of GRF, however this system is bound to some of the same restrictions as a laboratory, with high cost and requirement for additional equipment to log data collected by the system. Application of this type of system is also slightly more burdensome on the participant, due to wires and the data logger which must be with the participant. The group level accuracy and the portable nature of the RunScribe positions the system as an easy to apply measure of GRF, whereby more sophisticated laboratory equipment is required for direct measurement. GRF rate has been used within research studies to show association between cushioning properties present within footwear and reductions in GRF rate [18, 19]. Cushioning is a property within footwear that is highly sought after by a wearer, and is often subjectively related to footwear comfort [20]. High loading rates have previously been associated with lower limb stress fractures in runners [21], however this is limited to running and the lower loading rates associated with walking are not linked to stress injuries.

RunScribe shock variables differed in their agreement with the gold standard lab measurement. Impact shock showed the lowest difference between systems (mean differences of -2.69 –-5.28%) however showed low agreement (ICC = 0.189–0.474), far better than for braking shock (mean differences -78.10 –-100.32%) however with similar ICC results (ICC = 0.115–0.269). Due to total shock being derived from braking and impact shock it falls between their results in terms of reliability (mean differences -36.40 –-48.70% and ICC = 0.260–0.434). A previous study has compared impact shock measures of the RunScribe to peak positive acceleration (PPA) measured at the shank and shoe using an IMeasureU accelerometer showing overall correlation of r = 0.46, representing a moderate correlation between the reference accelerometer and the RunScribe [14]. Previous findings are consistent with current results whereby the RunScribe measures impact shock to be greater than a traditional IMU mounted on the shank. Reasoning for this difference is evidenced in previous research, which shows movement of the shoe independent of the foot and body can attenuate the shock experienced by the body [22]. Braking shock in the current research may be most affected by the notion of uncoupled motion of foot and shank, with the greatest differences present between the RunScribe and gold standard measurement system. During the time of peak impact shock the shank and foot move similarly, however peak braking shock is observed slightly later after foot contact, whereby the foot has experienced severe deceleration and is no longer in motion, whereas the shank has experienced deceleration resulting in a slowing down in forward progression of the shank. Another potential methodological constraint surrounding the Delsys accelerometer in the current investigation is the low sampling rate, using lower sampling rates for acceleration measurement has been associated with data loss within running research, it was however identified that lower sampling frequencies are required for walking speeds to provide accurate results [23]. Shock variables are becoming more prevalent in assessment of running footwear [24, 25] and insole products [26, 27] with manipulations in both products able to deliver shock absorption. Shock absorbing footwear is frequently posed as a method of injury prevention [28] with shock absorption also contributing to feelings of footwear comfort [29] leading to individuals seeking shock absorption within footwear. The RunScribe therefore has potential uses in footwear assessment in different domains for injury risk and understanding contributions of shock absorption to footwear comfort.

Pronation excursion shows moderate agreement (ICC = 0.604–0.616) and inconsistent mean differences (15.76%– 24.16%). Pronation velocity also displayed moderate agreement (ICC = 0.510–0.600) and larger mean differences (-24.33%– 27.94%). The RunScribe pronation variables are the only variables that have previously been compared to gold standard lab measures [13], however this was at running speeds. Agreement was mixed for pronation excursion with moderate agreement in the left limb (ICC = 0.57) but low agreement in the right limb (ICC = 0.40). Mean differences were also mixed, the left limb displayed a mean difference of -4.0° (-27.4%) and the right limb displaying a mean difference of 0.5° (3.6%). Good agreement was seen for pronation velocity in both left (ICC = 0.74) and right (ICC = 0.87) limbs, however mean differences were largely varied between limbs. Mean difference was much lower in the left limb at 8.6°/sec (Runscribe 1.9% greater than 3D motion) than in the right limb at 149°/sec (Runscribe 41% greater than 3D motion). Alongside more consistent difference to lab measures, the current results show more promise regarding the unidirectional difference compared to the lab for pronation excursion, with the RunScribe measuring both limbs lower than the lab. Pronation excursion is a key variable for many research studies examining orthotic intervention for reduction of frontal plane motion [30] and relationship between excessive pronation in runners and injury risk for medial tibial stress syndrome [31] and achilles tendinopathy [32]. Therefore, the RunScribe poses as an effective method for assessing this variable with a range of interventions within a real-world environment.

The present study has presented a comparison of the RunScribe to lab measures during walking for the first time, the current methodology does however contain some limitations. As previously mentioned, the difference in placement between the two measures of shock may have created some difference in measures resulting from attenuation seen at the shank not present at the foot. Shank mounting of the laboratory IMU was chosen as it is the gold standard for measurement of shock in research studies, therefore this protocol was followed in the current methods as the objectives of the study were to compare the measurements of the RunScribe to a gold standard laboratory system and methodology. Placing the laboratory IMU on the shoe would have provided greater agreement as the measurement systems would have been closer in proximity, however the method would then have been tailored to show greater agreement between the RunScribe and the laboratory system as opposed to reflecting standard approaches. No study to date, including the current study, has compared the repeatability of the RunScribe unit over a number of assessments or days. Further study with repeated measurements to provide more robust measures of accuracy, and proving the repeatability of the system would be key for the implementation of the RunScribe IMU within research testing interventions, and application within footwear or orthotic testing within the real-world environment. The RunScribe has however been previously applied in gait characterisation of healthy and injured runners [33], and a quantification of cumulative shock in runners to inform changes in training load [34]. The RunScribe is beginning to be used as a tool for real-world research, however further research should be completed assessing the sensitivity and repeatability of the RunScribe for the ability to detect differences between different footwear conditions and interventions, but also the ability to detect accurate individual differences within a participant sample.

## Conclusion

Current results display moderate agreement between the RunScribe IMU and the gold standard lab measurements for the majority of the variables. Some variables however show low agreement with traditional gold standard lab measurement. With varied agreement amongst variables, but also varied agreement between limbs the application of the RunScribe must be carefully considered when being used within intervention studies, and the comparison of data across studies. However, there has been emergence of the RunScribe IMU being used as a standalone tool within research in a variety of studies and this study expanded this yet further.

## Supporting information

S1 Data (XLSX)
